# Supplementary material for: Analysis of social combinations of COVID-19 vaccination: Evidence from a conjoint analysis
Source: PLoS One. 2022 Jan 21;17(1):e0261426. doi: 10.1371/journal.pone.0261426 (PMC8782289; doi:10.1371/journal.pone.0261426)
Supplement: S1 Fig — (PDF) [file pone.0261426.s002.pdf]

あなたにとって、以下の中で最も身近な存在は、どちらになりますか。

1つだけ選択してください。**そして何を選択されたか、よく覚えておくようにしてください**。

- 自分より年齢が上の同居家族（親など）
- 自分より年齢が下の同居家族（子どもなど）
- 職場の同僚
- 近所の人
- 配偶者
- 恋人・パートナー
- 友人

→

いまから、新型コロナウィルス感染症に対するワクチンの接種に関して、2種類の状況の中から、どちらか望ましいと思うものを1つ選択していただきます。

その際に、**「身近な存在」として前の質問で選択されたものを想定・思い起こしながら、ご回答ください**。

質問は5つ続きますので、ご回答くださいますようお願いいたします。また選択肢において、まれに同じ選択肢や、前の質問と同じものが提示されることがあります。その場合は「わからない」や「答えない」を選択し、回答を進めてください。

→

以下に2つの選択肢を挙げさせていただきます。

あなたにとって、以下の2つの状況のうちどちらが望ましいですか。

Choice 1とChoice 2のうち、どちらか一つをお選びください。

	Choice 1	Choice 2
**自分**	接種しない	副作用を確認して後から接種
**社会全般**	副作用を確認して後から接種	接種する
**身近な存在**	接種しない	接種しない

Choice 1

Choice 2

→

以下に2つの選択肢を挙げさせていただきます。

あなたにとって、以下の2つの状況のうちどちらが望ましいですか。

Choice 1とChoice 2のうち、どちらか一つをお選びください。

	Choice 1	Choice 2
**自分**	副作用を確認して後から接種	接種しない
**社会全般**	接種する	副作用を確認して後から接種
**身近な存在**	接種する	接種しない

Choice 1

Choice 2

→

以下に2つの選択肢を挙げさせていただきます。

あなたにとって、以下の2つの状況のうちどちらが望ましいですか。

Choice 1とChoice 2のうち、どちらか一つをお選びください。

	Choice 1	Choice 2
**自分**	副作用を確認して後から接種	接種する
**社会全般**	副作用を確認して後から接種	接種しない
**身近な存在**	副作用を確認して後から接種	副作用を確認して後から接種

Choice 1

Choice 2

→

以下に2つの選択肢を挙げさせていただきます。

あなたにとって、以下の2つの状況のうちどちらが望ましいですか。

Choice 1とChoice 2のうち、どちらか一つをお選びください。

	**Choice 1**	**Choice 2**
**自分**	副作用を確認して後から接種	副作用を確認して後から接種
**社会全般**	副作用を確認して後から接種	接種する
**身近な存在**	接種する	接種しない

Choice 1

Choice 2

→

以下に2つの選択肢を挙げさせていただきます。

あなたにとって、以下の2つの状況のうちどちらが望ましいですか。

Choice 1とChoice 2のうち、どちらか一つをお選びください。

	Choice 1	Choice 2
**自分**	接種しない	接種しない
**社会全般**	接種しない	副作用を確認して後から接種
**身近な存在**	接種する	接種しない

Choice 1

Choice 2

→
