# Supplementary material for: Analysis of social combinations of COVID-19 vaccination: Evidence from a conjoint analysis
Source: PLoS One. 2022 Jan 21;17(1):e0261426. doi: 10.1371/journal.pone.0261426 (PMC8782289; doi:10.1371/journal.pone.0261426)
Supplement: S4 Fig — (PDF) [file pone.0261426.s005.pdf]

Supplementary Materials: Figure 3

Analysis of Social Combinations of COVID-19 Vaccination:

Evidence from Conjoint analysis

November 17, 2021

Cross-bar plots of reasons for not vacctinating and watchers

Figure 1: Reasons for watchers by sex

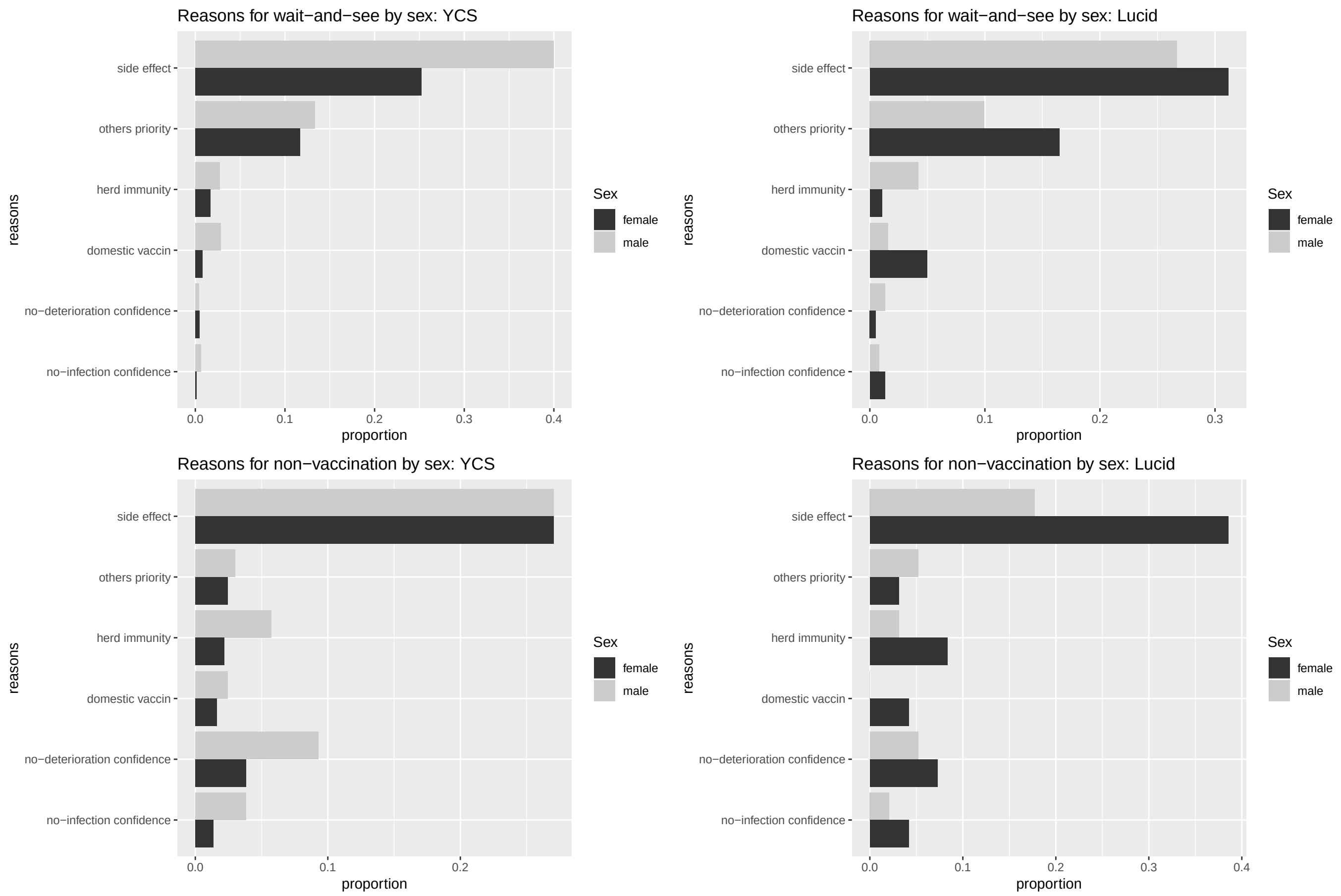

Note:Abbreviations: side effects=I want to see if there are any adverse reactions to the vaccine. Others priority=The product of the desired vaccine manufacturer is not available in Japan. Herd immunity=If others are inoculated first and herd immunity is established, there is no need to inoculate myself as soon as possible. No-deterioration confidence=Even if I am infected, it is unlikely that I will become seriously ill. No-infection confidence=I will not be infected. Infected=I have already been infected with COVID-19.

Figure 2: Reasons for watchers by education

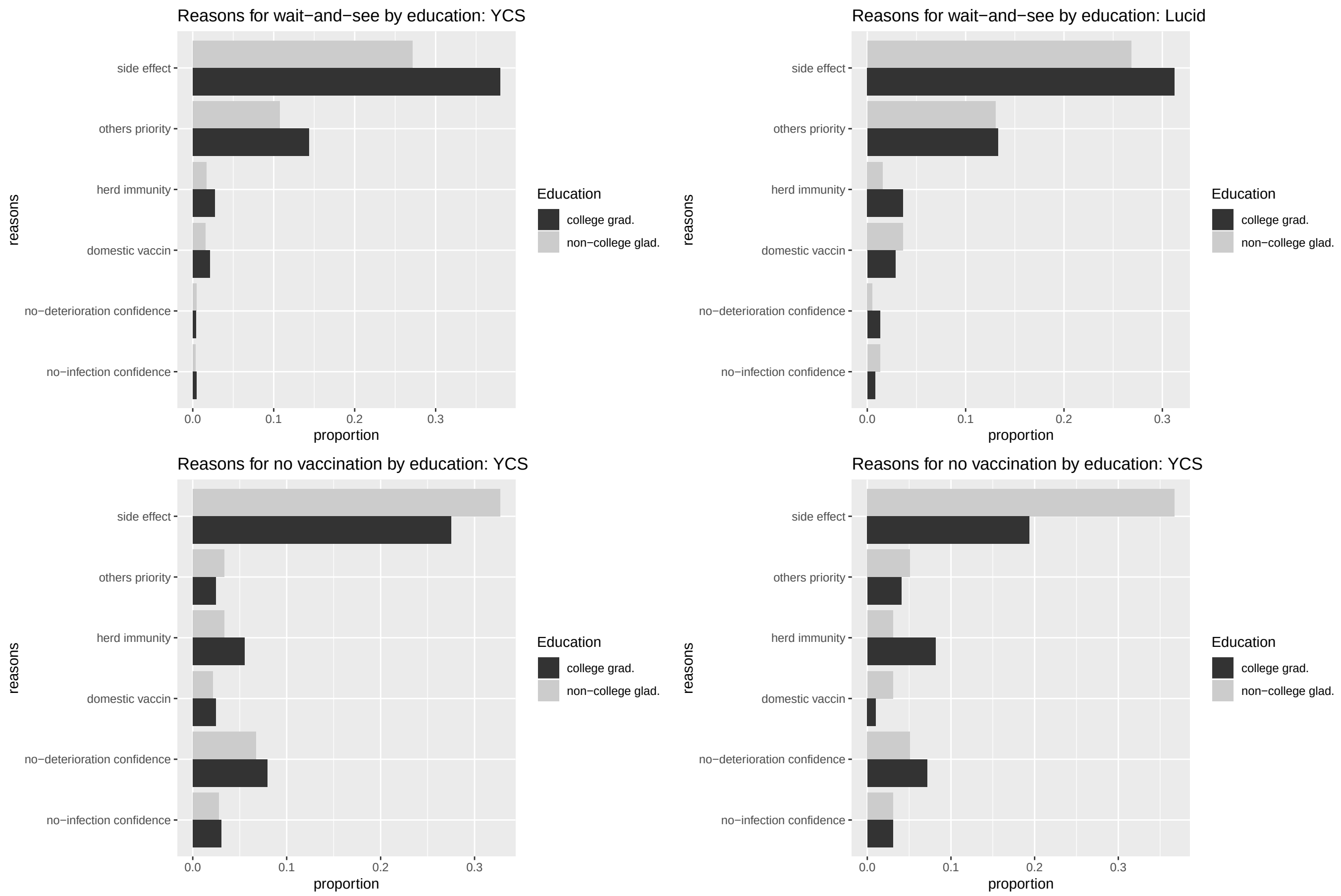

Note:Abbreviations: side effects=I want to see if there are any adverse reactions to the vaccine. Others priority=The product of the desired vaccine manufacturer is not available in Japan. Herd immunity=If others are inoculated first and herd immunity is established, there is no need to inoculate myself as soon as possible. No-deterioration confidence=Even if I am infected, it is unlikely that I will become seriously ill. No-infection confidence=I will not be infected. Infected=I have already been infected with COVID-19.
