# Supplementary material for: Analysis of social combinations of COVID-19 vaccination: Evidence from a conjoint analysis
Source: PLoS One. 2022 Jan 21;17(1):e0261426. doi: 10.1371/journal.pone.0261426 (PMC8782289; doi:10.1371/journal.pone.0261426)
Supplement: S8 Fig — (PDF) [file pone.0261426.s009.pdf]

Supplementary Materials: Table 2—4

Analysis of Social Combinations of COVID-19 Vaccination:

Evidence from Conjoint analysis

November 17, 2021

Descriptive Statistics and Demographic Composition

Table 1: Descriptive statistics of demography (Yahoo Crowd Sourcing)

	n	mean	sd	median	trimmed	mad	min	max	range	skew	kurtosis
age	2927	46.22	11.25	46.00	46.29	10.38	4.00	86.00	82.00	-0.05	0.01
gender (female=1)	2934	0.41	0.60	0.00	0.35	0.00	0.00	4.00	4.00	2.19	9.45
education (4year=1)	2896	0.57	0.50	1.00	0.59	0.00	0.00	1.00	1.00	-0.28	-1.92
income	2934	602.77	432.23	526.00	552.71	335.07	0.00	3000.00	3000.00	2.03	7.40

Table 2: Descriptive statistics of demography (Lucid)

	n	mean	sd	median	trimmed	mad	min	max	range	skew	kurtosis
age	994	44.59	16.15	44.00	44.19	19.27	8.00	86.00	78.00	0.16	-0.85
gender (female=1)	1024	1.53	0.55	2.00	1.51	1.48	1.00	4.00	3.00	0.61	0.78
education (4year=1)	1010	0.53	0.50	1.00	0.54	0.00	0.00	1.00	1.00	-0.14	-1.98
income	1024	869.09	654.91	684.50	777.85	493.71	0.00	3000.00	3000.00	1.32	1.54

Table 3: Comparison between survey demographic and national census (Yahoo Crowd Sourcing)

		Census	YCS	t-test results
Age				
	20-25	0.07	0.11	$t = 0$
	25-30	0.07	0.08	p-value = 1
	30-35	0.08	0.11	
	35-40	0.09	0.10	
	40-45	0.10	0.11	
	45-50	0.11	0.11	
	50-55	0.10	0.10	
	55-60	0.09	0.12	
	60-65	0.09	0.07	
	65-70	0.09	0.07	
	70-75	0.11	0.04	
Gender				
	Female	0.51	0.38	$t = 0.0003$
	Male	0.49	0.62	p-value = 0.9998
Education				
	Elementary	0.19	0.00	$t = 0$
	High Schoole	0.46	0.24	p-value = 1
	Two-year college	0.15	0.19	
	Four-year college and graduate school	0.20	0.57	
Income				
	0-100	0.07	0.02	$t = 0.136$
	100-200	0.13	0.07	p-value = 0.893
	200-300	0.14	0.12	
	300-400	0.13	0.12	
	400-500	0.10	0.12	
	500-600	0.09	0.14	
	600-700	0.07	0.08	
	700-800	0.06	0.08	
	800-900	0.05	0.08	
	900-1000	0.04	0.04	
	1000-1100	0.05	0.03	
	1100-1400	0.03	0.05	
	1400-1600	0.05	0.02	
	1600-1800	0.01	0.01	
	1800-2000	0.00	0.00	
	2000-	0.01	0.02	

Table 4: Comparison between survey demographic and national census (Lucid)

		Census	Lucid	t-test results
Age				
	20-25	0.07	0.02	$t = 0$
	25-30	0.07	0.04	p-value = 1
	30-35	0.08	0.08	
	35-40	0.09	0.12	
	40-45	0.10	0.16	
	45-50	0.11	0.21	
	50-55	0.10	0.15	
	55-60	0.09	0.11	
	60-65	0.09	0.07	
	65-70	0.09	0.04	
	70-75	0.11	0.02	
Gender				
	Female	0.51	0.50	$t = 0.003$
	Male	0.49	0.50	p-value = 0.998
Education				
	Elementary	0.19	0.02	$t = 0$
	High School	0.46	0.26	p-value = 1
	Two-year college	0.15	0.19	
	Four-year college and graduate school	0.20	0.53	
Income				
	0-100	0.07	0.02	$t = 0.168$
	100-200	0.13	0.04	p-value = 0.868
	200-300	0.14	0.09	
	300-400	0.13	0.09	
	400-500	0.10	0.09	
	500-600	0.09	0.11	
	600-700	0.07	0.06	
	700-800	0.06	0.08	
	800-900	0.05	0.05	
	900-1000	0.04	0.06	
	1000-1100	0.05	0.04	
	1100-1400	0.03	0.09	
	1400-1600	0.05	0.05	
	1600-1800	0.01	0.03	
	1800-2000	0.004	0.03	
	2000-	0.01	0.08	
