# Supplementary material for: Analysis of social combinations of COVID-19 vaccination: Evidence from a conjoint analysis
Source: PLoS One. 2022 Jan 21;17(1):e0261426. doi: 10.1371/journal.pone.0261426 (PMC8782289; doi:10.1371/journal.pone.0261426)
Supplement: S1 Questionnaire — (PDF) [file pone.0261426.s013.pdf]

"Survey on the Japanese Government's Performance Regarding COVID-19 Infections"

Request for your cooperation

Professor, School of Policy Studies, Kwansei Gakuin University
Kwansei Gakuin University

- This questionnaire is an academic survey conducted under a research grant from the Japan Society for Promotion of Science (Research Project No. 19K13615, "Research Project on the Relationship between Perceptions of Income Inequality and Political Support").

- This is not a survey for profit-making purposes or to support a specific political force. This survey is not for profit or to support any particular political party, nor will you suffer any disadvantage by cooperating with this survey.

- Please be aware that this survey will ask you about your awareness of the coronavirus disease of 2019 (COVID-19) infections. If you feel uncomfortable with the answers, you can stop answering at any time.

- The survey will also ask you about your income. If you feel uncomfortable with your answer, you can stop answering at any time.

- Some questions about politics and the government are difficult to answer. If you feel uncomfortable answering, you can stop at any time.

- All the answers obtained will be statistically processed so that the attributes of the respondents will not be known. All responses will be statistically processed so that the respondents' attributes will not be revealed, and no personal information will be identified.

- The results of the survey analysis will be actively disseminated to the general public through newspapers and other media. Thank you for your cooperation.

- Inappropriate responses degrade the quality of the survey results. Please do not answer any questions inappropriately. If you answer inappropriately, the survey may be

terminated.

- If you would like to start the survey, please click on the button saying “I agree with the purpose of this survey and the handling of personal information.” Then, click on the “>” or “Next” button on the bottom right to start the survey.

o I agree with the purpose of this survey and will cooperate in this survey. (1)
o I do not agree with the purpose of this survey and will not cooperate in this survey. (2)

Start of block: Face Sheet

Q2.1 Which of the following genders do you identify with?

o Female (1)

o Male (2)

o Neither (3)

o No answer (4)

Q2.2 Please indicate your current age.

Please enter your age in half-width numerals.

____

Q2.3 Which prefecture do you live in?

o Hokkaido (1)

o Aomori Prefecture (2)

o Iwate Prefecture (3)

o Miyagi Prefecture (4)

o Akita Prefecture (5)

o Yamagata Prefecture (6)

o Fukushima (7)

o Ibaraki (8)

o Tochigi Prefecture (9)

o Gunma (10)

o Saitama (11)

o Chiba (12)

o Tokyo (13)

o Kanagawa Prefecture (14)

o Niigata Prefecture (15)

o Toyama Prefecture (16)

o Ishikawa Prefecture (17)

o Fukui Prefecture (18)

o Yamanashi Prefecture (19)

o Nagano Prefecture (20)

o Gifu Prefecture (21)

o Shizuoka Prefecture (22)
o Aichi (23)
o Mie Prefecture (24)
o Shiga Prefecture (25)
o Kyoto Prefecture (26)
o Osaka (27)
o Hyogo Prefecture (28)
o Nara Prefecture (29)
o Wakayama Prefecture (30)
o Tottori Prefecture (31)
o Shimane Prefecture (32)
o Okayama (33)
o Hiroshima Prefecture (34)
o Yamaguchi (35)
o Tokushima (36)
o Kagawa (37)
o Ehime (38)
o Kochi (39)
o Fukuoka Prefecture (40)
o Saga Prefecture (41)
o Nagasaki Prefecture (42)
o Kumamoto Prefecture (43)
o Oita Prefecture (44)
o Miyazaki Prefecture (45)
o Kagoshima (46)
o Okinawa Prefecture (47)

Q2.4 Which of the following schools did you last graduate from? Please answer even if you have dropped out or are still enrolled as graduates.
o Elementary school (former elementary or high school) (1)
o High school (former junior high school) (2)
o Technical, junior, or special training college (3)
o Four-year university (4)
o Graduate school (5)
o Don’t know (6)

o No answer (7)

Q2.5 We would like to ask you about your marriage. In which of the following situations do you currently live?

o Married/partnered (1)

o Common-law/de facto marriage (4)

o Single (5)

o Divorced (6)

o Separated, etc. (not living with legal spouse) (7)

o Bereaved of spouse (8)

o Never married (9)

o Don’t know (10)

o No answer (11)

Q2.6 We would like to ask you about the composition of your family members who live with you. Which of the following family members do you currently live with? Please select all that apply.

▢ Children (1)

▢ Spouse (4)

▢ Domestic partner/de facto partner (5)

▢ Parents, or one of the parents (6)

▢ Spouse’s parent(s) or other parent(s) in law, or one of the parents in law (7)

▢ Relative(s) older than you (8)

▢ Relative(s) younger than you (9)

▢ Friend(s)/acquaintance(s) (10)

▢ Other (11) ____

▢ Live alone/single (2)

▢ Don’t know (3)

▢ No answer (12)

End of block: Face Sheet

Start of block: Prefectures, Cabinet/Governor/Prefectural Government Support

Q3.1 Do you support the cabinet of Yoshihide Suga?
o Support (1)
o Somewhat supportive (2)
o Neither supportive nor unsupportive (3)
o Somewhat unsupportive (4)
o Do not support (5)
o Don't know (6)
o No answer (7)

Q3.5 Many people seem to think that in the long run, they lean toward the X party. Please choose only one party.
o Liberal Democratic Party (1)
o Constitutional Democratic Party (2)
o Komeito (3)
o Japan Restoration Party (4)
o Japan Communist Party (5)
o National Democratic Party (6)
o Social Democratic Party (7)
o Reiwa Shinsengumi (8)
o Party to Protect the People from NHK (9)
o Party of Hope (10)
o Okinawa Socialist Popular Party (12)
o Team Shiga (13)
o Other (14) ____
o Don't know (15)
o No answer (16)

Q3.6 Do you consider yourself to be (or have you been) a particularly strong supporter of the following governments or political parties?

Please choose the option that best describes your level of support.

Particularly strong supporter (1)
Neither (2)

Not a particularly strong supporter (3)
Don’t know (4)
No answer (5)

Former Shinzo Abe administration (1) o o o o o o
Government of Yoshihide Suga (2) o o o o o o
Liberal Democratic Party (3) o o o o o o
Constitutional Democratic Party (5) o o o o o o
Komei Party (6) o o o o o o
Japan Restoration Association (7) o o o o o
Japan Communist Party (8) o o o o o o
National Democratic Party (9) o o o o o o
Social Democratic Party (10) o o o o o o
Reawa Shinsengumi (11) o o o o o o
Party to Protect the People from NHK (12) o o o o o o

End of block: Prefectures, Cabinet/Governors/Prefectural Government Support

Start of block: Individual- and Social-Oriented Economic Evaluation Including Questions Related to Income and Miscellaneous Deduction

Q4.1 How much is your household’s approximate annual income before taxes and take-home pay?

Income before taxes is generally referred to as “income at face value.” After taxes and social insurance premiums are subtracted from the face value income, the remainder is called take-home income.

Please specify income, including your and your spouse’s income, by sliding the following scale:

If all the answers are “30 million yen or more,” please select “3,000” (yen in million)

Annual household income before taxes (1)
Annual take-home income (2)

Q4.2 We would like to ask you some questions about your household's spending situation.

How much does your household spend each month on the following items?

Please select "100" if you spend more than 1 million yen.

Housing expenses (10,000 yen) *including mortgage (1)
Food expenses (10,000 yen) (2)
Education expenses (10,000 yen) (4)
Stock purchase and other investments (10,000 yen) (5)
Savings (10,000 yen) (6)

Q4.3 What do you think the government's economic policies mean to you?

Please rearrange the following items in the order in which you think they apply to you as economic policies.

Please note that the "Next" button will not appear until a certain amount of time has passed.

______ Prices (1)
______ Financial Reconstruction (2)
______ Stock Prices (3)
______ Tax System (4)
______ Social Security (5)
______ Personal Income (6)
______ Policy Rate (7)
______ Fiscal Stimulus (8)
______ Employment Measures (9)
______ Trade (10)
______ Foreign Exchange (11)
______ Reducing Inequality (12)

______ Economic Growth (13)

Q4.5 In each of the following areas, how would you rate the management of policies by the Japanese government?

“10” for “very good” ・ “1” for “very bad”

If you think the answer is “very bad,” please answer by sliding the scale to the nearest answer.

Please note that the “Next” button will not appear until a certain amount of time has passed.

_______ Prices (1)
_______ Fiscal Consolidation (2)
_______ Stock Prices (3)
_______ Tax System (4)
_______ Social Security (5)
_______ Personal Income (6)
_______ Policy Rate (7)
_______ Fiscal Stimulus (8)
_______ Employment Measures (9)
_______ Trade (10)
_______ Foreign Exchange (11)
_______ Reducing Inequality (12)
_______ Economic Growth (13)

Q4.7 We would like to ask you about your taxation for the previous fiscal year: fiscal year 2020.

Did your household claim miscellaneous loss deduction in your tax return for fiscal year 2020?

The deduction for miscellaneous losses means that a certain amount of income can be deducted when assets are damaged because of a disaster, theft, or embezzlement (from the National Tax Agency website: https://www.nta.go.jp/taxes/shiraberu/taxanswer/shotoku/1110.htm).

o Yes (1)
o No (2)

o Don’t know (3)

Show this question: If
I would like to ask you about your taxation for the previous fiscal year: fiscal year 2020. Did your household claim miscellaneous loss deduction in your tax return? = Yes

Q4.8 If you answered that you filed for miscellaneous loss deduction in fiscal year 2020, please answer the following question:

Which of the following is the reason why you claimed the miscellaneous loss deduction?

Please choose an answer from the following that applies the most to you.
o Disasters caused by anomalies in natural phenomena such as earthquakes, windstorms, floods, cold, snow, and lightning (1)
o Unusual disasters caused by humans, such as fires and explosions of explosives (2)
o Unusual disasters caused by living organisms such as pests (3)
o Theft (4)
o Embezzlement (5)
o Other (6) ____
o Don’t know (7)

Q4.9 I would like to ask you about your taxation for this year, 2021.

At this time, does your household plan to claim miscellaneous loss deduction on its tax return for fiscal year 2021?
o Yes (1)
o No (2)
o Don’t know (3)

Show this question: If
We would like to ask you about your taxation for this year, 2021. At this time, does your household plan to claim miscellaneous loss deduction on its tax return? = Yes

Q4.10 If you answered that you plan to file a miscellaneous loss deduction in fiscal year 2021,

please answer the following question:

Which of the following is the reason you are planning to claim the miscellaneous loss deduction?

o Disasters caused by natural phenomena such as earthquakes, windstorms, floods, cold, snow, and lightning (1)
o Disasters caused by abnormal human activities such as fire and explosions of explosives (2)
o Unusual disasters caused by living organisms such as pests (3)
o Theft (4)
o Embezzlement (5)
o Other (6) ____
o Don't know (7)

Q4.11 Have you used any of the following systems for COVID-19 infections that have spread since March 2020? Have you used the following systems or do you plan to use them?

Used (1)
Will use (2)
Will not use (3)
Don't know (4)
No answer (5)

Special flat-rate benefits (100,000 yen per individual) (1) o o o o o
Housing security benefit (2) o o o o o o
Sickness allowance (3) o o o o o o
Loan for welfare for daily life (small emergency fund) (4) o
Loan of welfare funds (general support funds) (5) o o o o o o
Livelihood security fund for job leavers (6) o
COVID-19 benefits (7) o o o o o o
Support and benefits for absence from work in response to COVID-19 infection (8) o
Subsidies for employment adjustment (9) o o o o o o
Subsidies for office rent (10) o o o o o o
Subsidies for projects to promote new business development (11) o
Subsidies for promotion of support, etc. (12) o

Business continuity support for businesses requesting suspension of operations (13) o o o
Special loans for COVID-19 infections (14) o o o o o o
Safety Net Guarantee No. 4 (15) o o o o o o
Safety Net Guarantee No. 5 (16) o o o o o o
Crisis-related guarantees (17) o o o o o o

End of block: Individual- and Social-Oriented Economic Evaluation Including Questions Related to Income and Miscellaneous Deduction

Start of Block: CJ Pre-Vaccine Questions

Q5.1 Which of the following individuals are the most familiar to you? Please select one option and memorize it.
Please choose only one, and try to remember what you have chosen.
o Family members living with you who are older than you (e.g., parents) (1)
o Family members living with you who are younger than you (e.g., children) (2)
o Colleagues at work (3)
o Neighbors (4)
o Lover/Partner (5)
o Spouse (6)
o Friends (7)

Q5.2 In the next question, you will be asked to select one of the two situations that you think is preferable regarding the COVID-19 vaccination.

In this case, please assume and recall the option you selected in the previous question as the most familiar.

There are five questions in total, so please make sure to answer all of them.

End of Block: CJ Pre-Vaccine Questions

Start of the block: CJ Vaccine

Q6.1 Here, there are two options offered.

Which of the following two situations do you prefer?

Please select Choice 1 or Choice 2.

Choice 1Choice 2${e://Field/F-1-1}${e://Field/F-1-1-1}${e://Field/F-1-2-1}${e://Field/F-1-2} }${e://Field/F-1-3}${e://Field/F-1-1-3}${e://Field/F-1-2-3}
o Choice 1 (1)
o Choice 2 (2)

Q6.2 Here, there are two options offered.

Which of the following two situations do you prefer?

Please select Choice 1 or Choice 2.

Choice 1Choice 2${e://Field/F-2-1}${e://Field/F-2-1-1}${e://Field/F-2-2-1}${e://Field/F-2-2} }${e://Field/F-2-3}${e://Field/F-2-1-3}${e://Field/F-2-2-3}
o Choice 1 (1)
o Choice 2 (2)

Q6.3 Here are two options for you to choose from.

Which of the following two situations is preferable to you?

Please choose one from among Choices 1 and 2.

Choice 1Choice 2${e://Field/F-3-1}${e://Field/F-3-1-1}${e://Field/F-3-2-1}${e://Field/F-3-2} }${e://Field/F-3-3}${e://Field/F-3-1-3}${e://Field/F-3-2-3}
o Choice 1 (1)
o Choice 2 (2)

Q6.4 Here, there are two options offered.

Which of the following two situations do you prefer?

Please select Choice 1 or Choice 2.

Choice 1Choice 2${e://Field/F-4-1}${e://Field/F-4-1-1}${e://Field/F-4-2-1}${e://Field/F-4-2} }${e://Field/F-4-3}${e://Field/F-4-1-3}${e://Field/F-4-2-3}
o Choice 1 (1)
o Choice 2 (2)

Q6.5 Here, there are two options offered.

Which of the following two situations do you prefer?

Please select Choice 1 or Choice 2.
Choice 1Choice 2${e://Field/F-5-1}${e://Field/F-5-1-1}${e://Field/F-5-2-1}${e://Field/F-5-2} }${e://Field/F-5-3}${e://Field/F-5-1-3}${e://Field/F-5-2-3}
o Choice 1 (1)
o Choice 2 (2)

End of the block: CJ Vaccine

Start of block: Negative Stimuli
Show this question: If
Which prefecture do you live in? = Hokkaido

Q7.1 In Hokkaido, where you live, Governor Naomichi Suzuki is currently in charge of the prefectural government.
As a result of the spread of the COVID-19, which has become noticeable since around March 2020, the number of people in Hokkaido who tested positive (cumulative total) for this infection was 17,445 as of midnight on February 1, 2021.

As of midnight on February 1, 2021, the number of people who tested positive (cumulative total) was 17,445, the number of people who are seriously ill (at present) is 12, and the

number of people who have died (cumulative total) is 602.

The situation is as follows. Compared with other prefectures, the number of people per capita in Hokkaido is quite high.

Contrastingly, in the neighboring prefecture of Aomori, the number of people who have tested positive (cumulative total) is 717, the number of people who are seriously ill (at present) is 2, and the number of people who have died (cumulative total) is 13.

… for the other 46 prefectures…

Q7.49
Due to the spread of COVID-19 since March 2020, the following situation has been observed in Japan as of midnight on February 1, 2021.

The number of people who have tested positive (cumulative total) for COVID-19 is 387,358, the number of people who are seriously ill (at present) is 975, and the number of people who have died (cumulative total) is 5,720.

Compared with other countries, in this situation, the spread of infection in Japan is progressing to a certain extent when considering the number of people per population.

Contrastingly, in the neighboring country of South Korea, the number of people who tested positive (cumulative total) is 78,508, the number of people who are seriously ill (at present) is 225, and the number of deaths (cumulative total) is 1,425.

Compared with the neighboring country of South Korea, the spread of the infection has progressed in Japan to a considerable extent and has failed to be controlled in terms of the number of people per population.

End of block: Negative Stimulus

Start of block: Positive Stimulus

Show this question: If
Which prefecture do you live in? = Hokkaido

Q8.1 In Hokkaido, where you live, Governor Naomichi Suzuki is currently in charge of the provincial government.
As a result of the spread of a COVID-19 pneumonia, which has become noticeable since around March 2020, the number of people who have tested positive (cumulative) for this infection in Hokkaido as of midnight on February 1, 2021, is as follows.

As of midnight on February 1, 2021, the number of people who have tested positive (cumulative total) is 17,445, the number of people who are seriously ill (at present) is 12, and the number of people who have died (cumulative total) is 602.

The situation is as follows. Compared with other prefectures, in this situation, the spread of infection has been controlled to some extent in terms of the number of people per population.

In contrast, in the neighboring prefecture of Aomori, the number of people who have tested positive (cumulative total) is 717, the number of people who are seriously ill (at present) is 2, and the number of people who have died (cumulative total) is 13.

Compared with other prefectures, Aomori Prefecture is considered to have failed to control the spread of the disease to a much greater extent in terms of the number of people per population.

… for the other 46 prefectures…

Q8.49

With the spread of COVID-19 pneumonia since March 2020, the following situation has been observed in Japan as of midnight on February 1, 2021.

The number of people who have tested positive (cumulative total) for COVID-19 is 387,358, the number of people who are seriously ill (at present) is 975, and the number of people who have died (cumulative total) is 5,720.

Compared to other countries, the number of people per population has been successfully

controlled to some extent.

Contrastingly, in the neighboring country of South Korea, the number of people who have tested positive (cumulative total) is 78,508, the number of people who are seriously ill (at present) is 225, and the number of people who have died (cumulative total) is 1,425.

Compared with the neighboring country of South Korea, Japan succeeded in controlling the spread of infection to a large extent in terms of the number of people per population.

End of block: Positive Stimulus

Start of block: Control Group

Q9.1
The following is the Japan Meteorological Agency's explanation of greenhouse gases. Please read it.
(URL: https://www.data.jma.go.jp/cpdinfo/chishiki_ondanka/p04.html)

The main greenhouse gases that have increased due to human activities are carbon dioxide, methane, nitrous oxide, and chlorofluorocarbon gases.

Carbon dioxide is a greenhouse gas with the greatest impact on global warming. Large amounts of carbon dioxide are released into the atmosphere through the consumption of coal and oil and the production of cement. Additionally, forests that absorb carbon dioxide from the atmosphere are decreasing. Hence, the amount of carbon dioxide in the atmosphere is increasing every year.

Methane is the second largest greenhouse gas after carbon dioxide in terms of its impact on global warming. Methane is produced when dead plants decompose in wetlands, ponds, and rice paddies. It is also present in livestock burps. Moreover, methane is produced during the extraction of natural gas.

End of block: Control Group

Start of block: Re-Question (Governor/Prefectural Support)

Q10.1 I would like to ask you the following again.

Do you support the Yoshihide Suga cabinet?

Please note that no bonus will be given if you answer “Don’t know” after this.
o I support (1)
o Somewhat supportive (2)
o Neither supportive nor unsupportive (3)
o Somewhat unsupportive (4)
o Do not support (5)
o Don’t know (6)
o No answer (7)

Q10.2
I would like to ask you the following question again.
The following are the names of some organizations and people involved in politics.
To what extent do you support the overall political management by the following organizations or people? To what extent do you support the overall political management by the following organizations or people, or do you not support them?

Approve (1)
Somewhat approve (2)
Neither approve nor disapprove (3)
Somewhat disapprove (4)
Do not disapprove (5)
Do not know (6)
No answer (7)

Japanese government (1) o o o o o o o
Show this option: If
Which prefecture do you live in? = Hokkaido
Naomichi Suzuki, Governor of Hokkaido (2) o o o o o o o o

…for the other 46 prefectures…

Show this option: If
Which prefecture do you live in? = Hokkaido
Hokkaido Politics (49)

…for the other 46 prefectural administrations.

End of block: Re-Question (Governor/Prefectural support)

Start of block: Intention to be Vaccinated

Q11.1 Vaccination against COVID-19 infections began in February 2021.

Please choose the answer that most closely matches your opinion regarding the vaccine for COVID-19 infection from among the following.
o Vaccinate against COVID-19 infection (1)
o Vaccinate later rather than earlier (2)
o Do not vaccinate against COVID-19 infection (3)
o Don't know (4)
o No answer (5)

Show this question: If
Vaccination against COVID-19 infection began in February 2021. Please choose the answer that most closely matches your opinion regarding the vaccine for the COVID-19 infection from among the following. = Vaccinate later rather than earlier.

Q11.2 If you answered "Vaccinate later rather than earlier," we would like to ask you the following.

For which of the following reasons would you prefer to be vaccinated against COVID-19 infections at a later date?
o To check for adverse reactions to the vaccine (1)
o Because the product of the manufacturer of the vaccine I want is not available in Japan (2)

o Because I do not need to be vaccinated soon since others were vaccinated first and herd immunity is established (3)
o Because I am unlikely to be infected (4)
o Even if I am infected, I will not get seriously ill (9)
o Because other people should be vaccinated before me (5)
o Because I have already been infected with COVID-19 (10)
o Other (6) ____
o Don’t know (7)
o No answer (8)

Show this question: If
Vaccination against COVID-19 infection began in February 2021. Please choose the answer that most closely matches your opinion regarding the vaccine for COVID-19 infections from among the following. = Do not vaccinate against COVID-19 infection.

Q11.3 If you answered “Do not vaccinate against COVID-19 infection,” we would like to ask you the following.

For the following reasons, do you think you will not be vaccinated against COVID-19?
o Because I am afraid of adverse reactions to the vaccine (1)
o Because the product of the manufacturer of the vaccine I want is not available in Japan (2)
o Because I do not need to be vaccinated soon since others were vaccinated first and herd immunity is established (3)
o Because I am unlikely to be infected (4)
o Because I am not likely to become seriously ill even if I am infected (5)
o Because other people should be vaccinated before me (6)
o Because I have already been infected with COVID-19 (7)
o Other (8) ____
o Don’t know (9)
o No answer (10)

End of block: Intention to be vaccinated

Start of block: Debriefing

Q12.1 This is the last screen of the survey, and it provides the "re-explanation of the purpose of the survey" and "final confirmation of the intention to send the results."

In this survey, we asked you about your attitude toward COVID-19. We would like to express our sincere gratitude for your cooperation. We hope that you will find this information useful.

- Housing security benefit
- Sickness allowance
- Loan for daily life welfare fund (emergency small-lot fund)
- Welfare fund loan (general support fund)
- Livelihood security fund for job leavers
- Subsistence payment
- COVID-19 disease leave support and benefits
- Employment adjustment subsidies
- Subsidies for office rent
- Subsidies for promotion of new business development, etc.
- Subsidies for promotion of support, etc.
- Support for business continuity for businesses requesting leave of absence
- Special loan for COVID-19 infection
- Safety Net Guarantee No. 4
- Safety Net Guarantee No. 5
- Crisis-related guarantees

In this survey, a "comparison of the infection status of COVID-19 between Japan and its neighboring country, South Korea" was presented in some cases. In this survey, information on "a comparison of the infection situation between Japan and its neighboring country, South

Korea, regarding COVID-19" was presented in some cases, and some expressions, including the statement "when considering the number of people per population" could be judged as exaggerating the infection situation in South Korea. Although the information was exaggerated for academic purposes, the actual situation is different, and we would appreciate it if you would bear this in mind. However, if you feel uncomfortable or have any doubts, you can choose "I do not agree with the purpose of this survey, so I will not send my response results."

Furthermore, this survey was designed to ask you about your psychological attitude toward the issue of COVID-19 infection. By examining your intentions regarding vaccination, in particular, this survey intends to identify the most desirable vaccination situation in society. You may have felt uncomfortable or that some of the content contained exaggeration. If this is the case, you can choose "I do not agree with the purpose of this survey, so I will not send my response results."

If you do not agree with the purpose of this survey, you may choose not to send your response.
o I agree with the purpose of this survey and will send my response results. (1)
o I do not agree with the purpose of this survey, so will not send my response results. (2)

Skip to the next: End of Survey Condition. This is the last screen of the survey that provides the "re-explanation of the purpose of the survey" and "final confirmation of your intention to send the response results." In this survey, we asked you about your awareness of COVID-19 infections. = I do not agree with the purpose of this survey, so I will not send my response results.

End of block: Debriefing
