# Supplementary material for: Analysis of social combinations of COVID-19 vaccination: Evidence from a conjoint analysis
Source: PLoS One. 2022 Jan 21;17(1):e0261426. doi: 10.1371/journal.pone.0261426 (PMC8782289; doi:10.1371/journal.pone.0261426)
Supplement: Attachment — Submitted filename: response_letter_plosone2021201.docx [file pone.0261426.s015.docx]

Reviewer #2

I would like to thank you for your second detailed review of this paper and for your valuable suggestions. I have responded to each comment as follows and would appreciate your confirmation.

**You were using "I" but at some points in the method section, you were using "our" and "us". Please, be consistent.**

I have ensured that all instances of “us” and “our” were deleted in this newly revised version.

**Methods**

**Design of conjoint analysis: This sentence may not be complete, “According to the above conjoint analysis settings, I set my design of conjoint as in Table 1 and Figure 1, and Figure shows an example of the conjoint screen.” I think ". . . Figure shows an example of the conjoint screen." should have a number as ". . . Figure 2 shows an example of the conjoint screen."**

Thank you for pointing this out. The figure number was indeed missing, so I have added it accordingly.

**Results**

**Lines 137-138: ". . . as predicted, the percentage exceeded 50% in all surveys (Figure )." The figure is missing a number.**

I would like to thank you for your careful review of this as well. I have added the figure number accordingly. I have also embedded each percentage value in the bar graph in the figures. I hope that the readability of the figures has improved.

**Conclusion**

**The information you provided as a limitation does not reflect a study limitation. Your limitation should focus on the measures, study design and sampling, analytical procedures, etc. For instance, this is a cross-sectional study, so you could not establish causality and temporality.**

I would like to thank you for your kind remarks. One of the limitations of this study is that the data were not panel data consisting of several waves, the unit specific effects were not controlled, and there was a difficulty in handling covariates. I would appreciate it if you could confirm this (l.204–l.212).
